# Pathogenicity Prediction of Missense Variations in Hereditary Cancer Genes

**DOI:** 10.3390/ijms27052453

**Published:** 2026-03-07

**Authors:** Cemaliye B. Akyerli, Gizel Gerdan, Alper Bülbül, Hilal Keskin-Karakoyun, Şirin K. Yüksel, Emel Timucin

**Affiliations:** 1 Department of Medical Biology, School of Medicine, Acibadem Mehmet Ali Aydinlar University, Istanbul 34638, Türkiye; 2 Department of Genome Studies, Graduate School of Health Sciences, Acibadem Mehmet Ali Aydinlar University, Istanbul 34638, Türkiye; gizel.gerdan@live.acibadem.edu.tr; 3 Department of Biostatistics and Bioinformatics, Graduate School of Health Sciences, Acibadem Mehmet Ali Aydinlar University, Istanbul 34638, Türkiye; 4 Department of Biochemistry and Molecular Biology, Graduate School of Health Sciences, Acibadem Mehmet Ali Aydinlar University, Istanbul 34638, Türkiye; keskin-hilal@hotmail.com (H.K.-K.);; 5 Department of Molecular Biology and Genetics, Gebze Technical University, Kocaeli 41400, Türkiye

**Keywords:** hereditary cancer, pathogenicity classification, VUS, AlphaFold2

## Abstract

HerCanPred, a machine-learning-based pathogenicity classifier specifically optimized for 63 cancer-predisposition genes, was developed to improve the interpretation of missense variants in hereditary cancer syndromes. This model integrates sequence conservation with structural features derived from AlphaFold2 (AF2) structures. HerCanPred achieved a strong performance, outperforming 23 established predictors. SHAP analysis identified AF2-derived structural features, specifically local pLDDT confidence scores and relative solvent accessible area, as the strongest predictors of variant impact. Benchmarking the strengths and limitations of HerCanPred against existing methods showed that misclassification of pathogenic variants was concentrated in disordered and surface-exposed regions, whereas benign failures were more broadly distributed. HerCanPred and three established predictors were also applied to over 57,000 variants of uncertain significance (VUS) from the same gene set. Notably, 166 VUS were reassigned as pathogenic and 75 as benign, with an enrichment of the *NF1*, *FH*, and *MLH1* genes. By combining gene-specific training with 3D structural information, HerCanPred provides a robust framework for reducing diagnostic uncertainty. Our findings demonstrate that targeted, structure-aware tools can contribute to resolving VUS, providing a rational basis for systematic variant reinterpretation and more informed medical management in hereditary cancer care.

## 1. Introduction

Approximately 10% of malignancies are caused by hereditary cancer syndromes (HCSs) [1]. Recognizing these often genetically heterogeneous syndromes, which confer an increased risk of cancer, is clinically important [2]. In particular, many genes involved in the cell cycle and DNA repair harbor pathogenic variants [3]. Most HCSs exhibit autosomal-dominant inheritance, conferring a 50% risk of transmission to offspring [1]. This dominant inheritance refers to the clinical transmission of cancer predisposition within families and does not imply that all germline variants identified in these genes are pathogenic. Indeed, large numbers of polymorphic and rare germline variants detected in the population remain of uncertain clinical significance. Distinguishing such variants from true disease-causing alterations represents a major challenge in hereditary cancer genetics. As new methods for interpreting genetic data emerge, approaches to diagnosis, risk assessment, prognostication, and individualized treatment for hereditary cancers require continual updating [4].

Multiple hereditary cancer gene panels are available and generally follow similar frameworks. Genetic testing panels used to determine hereditary cancer predisposition generally include genes associated with DNA repair, cell cycle, homologous recombination, and cellular senescence/apoptosis [5,6,7,8]. Nowadays, a significant amount of genetic data has been processed and collected in databases, including different missense variants related to HCSs [9]. Therefore, we now have access to a vast amount of knowledge on genetic variants associated with HCSs present in the general population.

Variants are classified into five categories by the American College of Medical Genetics and Genomics/Association for Molecular Pathology (ACMG/AMP). These are benign, likely benign, variants of uncertain significance (VUS), likely pathogenic, and pathogenic based on multiple evidence types [10]. Distinguishing pathogenic from benign variants is central to hereditary disease testing [11]. Accurate variant classification matters because likely pathogenic or pathogenic results are actionable for diagnosis, prognosis, treatment/prevention, and family risk assessment [12,13]. The effectiveness of these classification guidelines and thresholds remains under active study [14,15,16].

Preliminary reports often fall short of a definitive label, so variants are likely to be initially classified as VUS [17]. Over 40% of clinically reported variants in ClinVar are VUS or have conflicting classifications [18,19]. The misclassification of VUS can influence prophylactic surgery decisions and risk prediction. To refine VUS interpretation, laboratories integrate functional assays with curated clinical databases and predictive algorithms, enabling faster validation and clinical annotation. These efforts are critical to reclassifying and reducing the number of VUS variants [20]. In silico tools using sequence- and/or structure-based features are increasingly applied to (re-)classify variants [21,22]. However, existing computational approaches often struggle with hereditary cancer genes, where the clinical impact of specific variants remains particularly uncertain.

We merged two widely used hereditary cancer panels (26- and 59-gene) to define a set of 63 genes and compiled ClinVar missense variants for these genes. Using this exclusive dataset, we trained a tailored variant effect classifier, Hereditary Cancer Predictor (HerCanPred), relying on both sequence and structure-based features from 63 genes. We then applied HerCanPred, alongside established predictors, to over 57,000 VUS from the same genes to prioritize computational reclassifications, resolving a small subset of VUS cases. Finally, we benchmarked performance and evaluated the strengths and limitations of HerCanPred relative to existing methods.

## 2. Results

### 2.1. Protein–Protein Interactions (PPIs) Within Hereditary Cancer Panels

**Figure 1:**
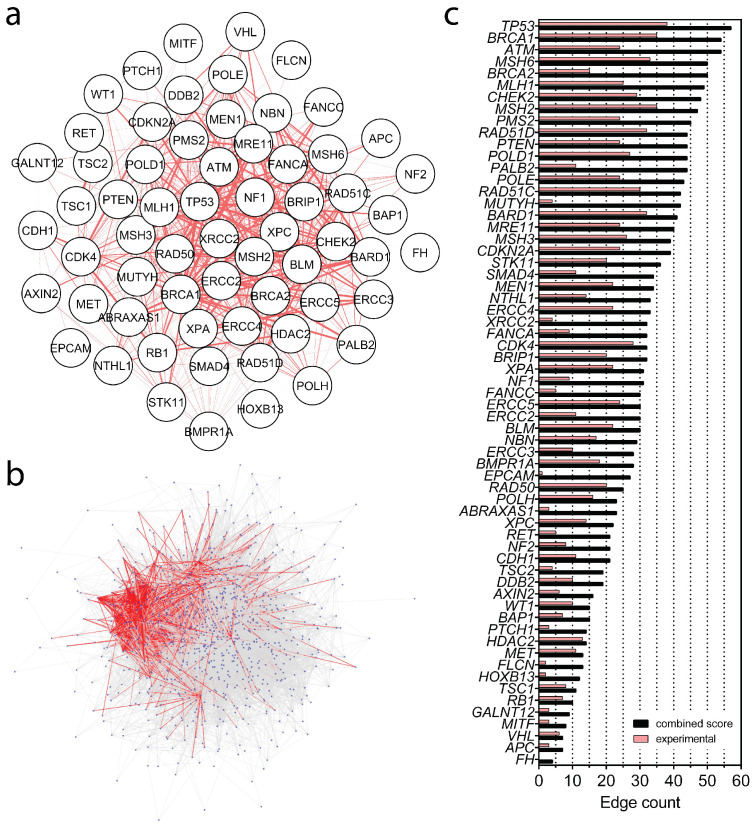
Protein–protein interaction (PPI) networks of (**a**) 63 genes from 2 panels and (**b**) 902 genes from 4 panels are shown, wherein the edge attribute is set as the score of the experimentally detected interactions from STRING DB. The connections between 63 nodes are colored red and 902 nodes are depicted as blue dots in (**b**). (**c**) Edge count of each gene in (**a**) is plotted; black bars represent edge counts based on the combined score obtained from text-mining/databases, experiments and co-expression/co-occurrence statuses of genes, whilst red bars show the counts of experimentally determined interactions.

We analyzed 63 hereditary-cancer-associated genes to characterize their interaction network and quantify their connectivity within PPI networks (Figure 1a). Several genes, most notably *TP53*, *BRCA1*, *ATM*, and *NF1*, function as highly connected hubs, consistent with their central roles in DNA damage response, DNA repair, and cell-cycle regulation [7]. To place this subset in a broader context, we assembled a larger superset of 902 distinct genes by combining cancer-related genes reported in OMIM, HPO, and ClinVar databases (see Supplementary Materials), including the 26- and 59-gene panels, and mapped all pairwise interactions (Figure 1b). Over 21,000 PPIs were identified for these 902 genes, of which 5088 edges involved at least one of the genes in the 63-gene set. Although the 63 genes constitute only ∼7% of the 902-gene universe, they account for nearly one quarter of all interactions in the PPI network, highlighting their significant contribution to overall connectivity (Figure 1b). Figure 1c reports gene-level edge counts, comparing combined interaction scores (dark bars) with experimentally supported interactions (light bars). *TP53*, *BRCA1*, *ATM*, *MSH6*, and *BRCA2* exhibit the highest degree, reinforcing their prominence in cancer predisposition networks. Overall, these analyses indicate a tightly interconnected network in which a small set of genes captured a large fraction of the observed connectivity. Accordingly, this set of 63 genes was used to develop targeted variant effect predictors for hereditary cancer genes.

### 2.2. Missense Variants of 63 Hereditary Cancer Genes

**Figure 2:**
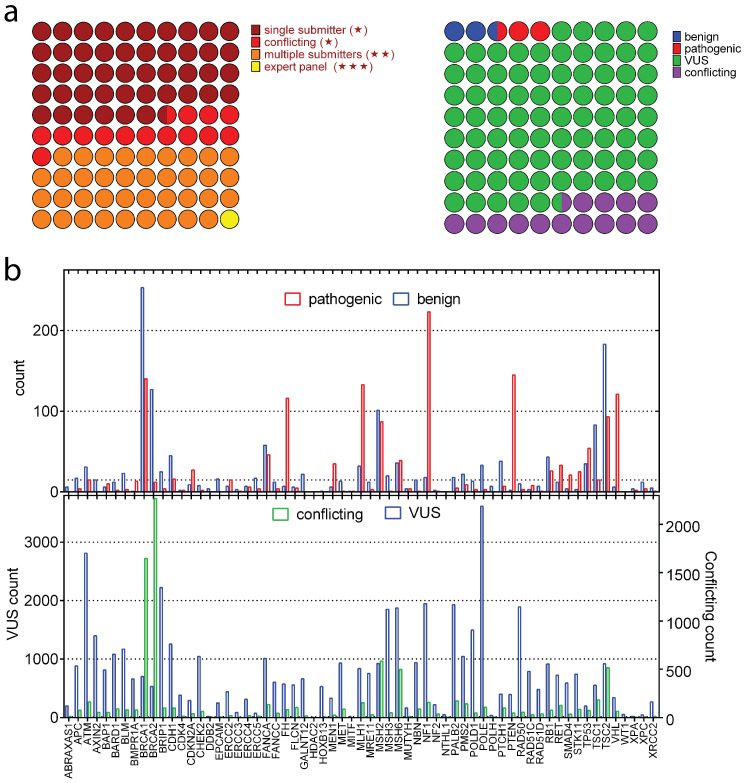
Overview of missense variants collected from 63 genes. (**a**) Breakdown according to ClinVar review stars and clinical significance labels. (**b**) Distribution of clinical significance of variant classifications across the 63 genes, pathogenic versus benign (**top**) and VUS versus conflicting interpretations (**bottom**).

The integration of ClinVar variants with UniProt and the AlphaFold Protein Structure Database yielded 60,785 clinically annotated variants from an initial pool of 96,000 spanning 63 genes. Classification by submitter reliability indicates a predominance of single-submitter entries relative to expert-panel-reviewed variants (Figure 2a). The clinical significance distribution shows that most variants are designated as VUS (green), followed by conflicting interpretations (purple), with benign (blue) and pathogenic (red) categories representing smaller fractions (Figure 2b). Gene-level summaries showed an unbalanced distribution of clinical significance labels across 63 genes (Figure 2b—top). Pathogenic variants were particularly enriched in genes such as *BRCA1*, *FH*, *NF1*, *PTEN*, *TSC2* and *VHL*, whereas others, such as *BRCA1*, *BRCA2* and *TSC2*, exhibit a higher proportion of benign variants. Similarly, a notable heterogeneity was observed in the distribution of variants with VUS and conflicting interpretations (Figure 2b—bottom). A dominance of VUS labels was observed compared to conflicting interpretations except for *BRCA1* and *BRCA2* genes, which showed over 2500 variants with conflicting pathogenicity labels. Particularly, *ATM*, *BRIP1* and *POLE* had over 2000 VUS labels. Large fractions of variants with VUS and conflicting interpretations in most of the genes underline a dominance of unknown/conflicting labels within these hereditary genes, emphasizing the need for additional efforts to resolve their significance.

Overall, from this large variant set, we assembled 3091 variants across 63 genes annotated as pathogenic or benign and used them for model development. Variants labeled as VUS (48,739) or with conflicting interpretations (8923) were withheld from training and reserved for subsequent reclassification analyses.

### 2.3. Development of a Classifier for Variants in Hereditary Cancer Genes

Following a rigorous model selection using nested cross-validation and Bayesian hyperparameter optimization, we trained two machine learning classifiers, which we collectively refer to as the Hereditary Cancer Predictor (HerCanPred). Of the two tested models (random forest classifier: HerCanPred_*rf*_, XGBoost classifier: HerCanPred_*xgb*_), HerCanPred_*rf*_ was identified as the best model for this classification task according to all evaluated metrics (Table 1).

**Figure 3:**
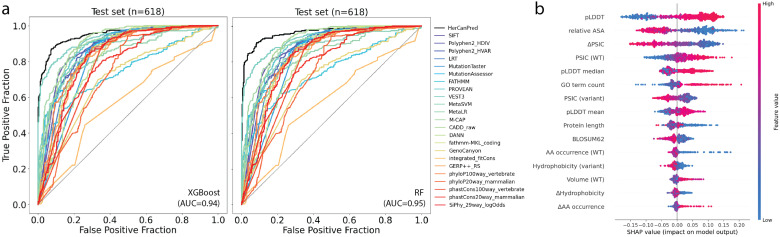
Performance and feature importance of HerCanPred pathogenicity classifier: (**a**) ROC curves comparing the performance of HerCanPred (black curves) against other pathogenicity prediction tools on the test set. (**b**) SHAP-based feature importance is shown as a ranked summary of the relative contributions of top 10 features of the HerCanPred_*rf*_ model.

To understand which features were most influential in the HerCanPred_*rf*_, we conducted SHAP analysis (Figure 3b). The analysis revealed two structural features, namely, the pLDDT score and rASA of the variant position, as the most important features. While low pLDDT values, which originally indicate low confidence in the predicted local protein structure [23], were strongly associated with a pathogenic outcome, high rASA values, which typically represent disordered regions, reflect a signal for pathogenicity. Trailing these two, the third most important feature is the PSIC score difference (ΔPSIC). A high difference in PSIC score upon variation, which signifies a drastic change at a conserved position, pushed the prediction toward pathogenicity, whilst a low score difference signaled neutral variations. Other influential features included evolutionary conservation metrics, such as PSIC scores for wild-type and variant amino acids. Furthermore, protein-based features such as protein length and GO term counts appeared among the top 10 important features. Overall, the HerCanPred_*rf*_ model learned to identify pathogenic variants by integrating information from structural instability and evolutionary constraint.

To assess the relative contribution of feature subsets, we compared the full HerCanPred_*rf*_ model against a reduced version (HerCanPred_*rf*___*Red.*_) that excluded gene-level and annotation-density features such as GO term counts and protein length to focus exclusively on sequence- and structure-based variant features. The ablation analysis demonstrated that the variant-specific features capture the majority of the pathogenicity signal, with the reduced model achieving an AUC of 0.93 (Table 2). The addition of gene/protein-level features provided only a moderate performance enhancement (ΔAUC = 0.03). This finding suggested that while this broader gene-level information, such as protein length, offers supplemental information, the main predictive capability is driven by localized variant characteristics.

### 2.4. Performance Assessment of HerCanPred Against Established Predictors

The performance of our two HerCanPred models was benchmarked against a comprehensive set of 23 variant effect predictors. The comparative performance metrics, including the AUROC, F1 score and MCC, are given in Table 2. Both HerCanPred models achieved the best predictive performance, outperforming established tools such as SIFT, PolyPhen2, and MetaSVM, with a notably higher true-positive rate across all false-positive rates (Figure 3a). This highlights their robustness and reliability for predicting pathogenic variants.

Our models, HerCanPred_*rf*_ and HerCanPred_*xgb*_, demonstrated an improved classification performance over existing methods for hereditary cancer variants. Specifically, HerCanPred_*rf*_ achieved an AUROC of 0.963 (95% CI [0.948, 0.976]), significantly outperforming all 23 compared methods. It also produced a superior F1 score of 0.91 and an MCC of 0.81 when compared with the rest of the predictors. The HerCanPred_*xgb*_ model also showed a high performance, securing the second-highest scores with an AUROC of 0.95, an F1 score of 0.90, and an MCC of 0.79. These results represent a marked advance over other leading tools for variant effect prediction, particularly for HerCanPred_*rf*_. For comparison, the next best-performing tool by AUROC was VEST3 (0.93), while tools like SIFT and PolyPhen2-HDIV achieved considerably lower F1 scores (0.81) and MCCs (0.60). This robust performance underscores the predictive power of HerCanPred for classifying hereditary cancer variants.

### 2.5. Performance Highlights and Failure Modes of HerCanPred_rf_

**Figure 4:**
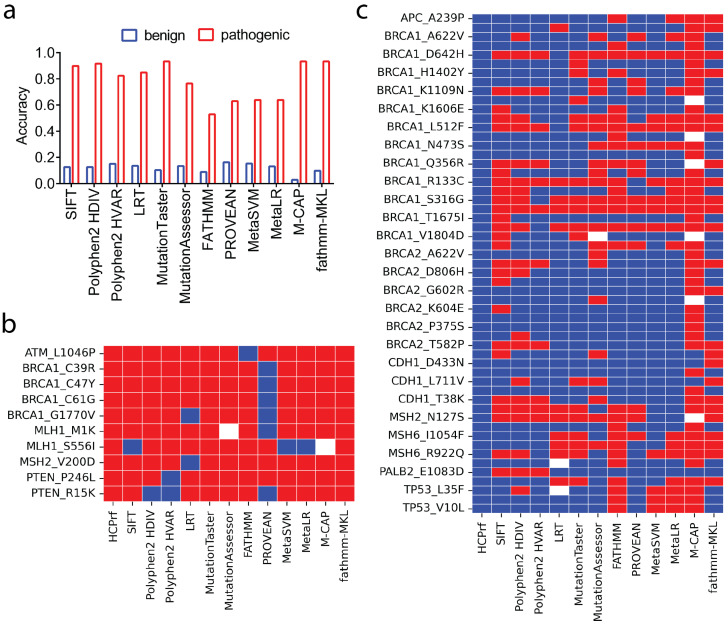
Performance evaluation of 12 predictors on a set of variants (pathogenic *n* = 275, benign *n* = 1110) for which the HerCanPred_*rf*_ predictor correctly classified all benign and pathogenic labels. (**a**) Accuracy of the predictors in classifying benign (blue) and pathogenic (red) variants. (**b**,**c**) provide heatmaps showing the performance of each predictor for each variant.

To dissect the performance gap between HerCanPred_*rf*_ and other predictors, we evaluated the performance on a subset of three-star-review variants correctly predicted by HerCanPred_*rf*_. We analyzed the performance of 12 predictors, which produced a binary outcome (Figure 4a). For pathogenic variants, per-class accuracies (sensitivity and specificity) were consistently high for each predictor (Figure 4a—red). Essentially, Mutation Taster, M-CAP and fathmm-MKL correctly predicted almost all pathogenic variants, closely trailing HerCanPred_*rf*_. We further showed predicted labels for the set of pathogenic variants with a three-star review (expert panel) (Figure 4b). In line with their overall high sensitivity, all 12 predictors correctly labeled the expert-reviewed pathogenic variants. Although FATHMM showed the lowest accuracy for pathogenic variants (Figure 4a), it performed much better for three-star-review variants, missing only one variant from *ATM* (Figure 4b). Similarly, MetaSVM and MetaLR missed only a single pathogenic variant, yielding a higher accuracy for three-star-review variants than for all variants, including those with a single star or two stars. Notably, PROVEAN showed the weakest performance on both all-star- and three-star-review pathogenic variants.

For benign variants that were correctly predicted by HerCanPred_*rf*_, we reported a substantially lower specificity for all predictors (Figure 4a—blue). When focused on only three-star-review benign variants, a consistently low performance was observed for all 12 predictors (Figure 4c). One notable point was the dominance of *BRCA1* and *BRCA2* benign variants with three stars. Particularly, *BRCA1* variants were largely missed by the comparator predictors, while *BRCA2* benign variants were correctly captured by a number of predictors except for M-CAP and fathmm-MKL, which largely missed benign variants from both *BRCA1* and *BRCA2*. These per-variant views highlight that errors are not confined to a small subset of tools or genes but are distributed across predictors, with several methods sharing similar failure modes on specific benign variants. Together, these results indicate that most existing predictors prioritize sensitivity over specificity, performing well on pathogenic variants but failing on benign variation. This analysis underscores the power of HerCanPred_*rf*_ for reducing false positives while maintaining high sensitivity.

**Figure 5:**
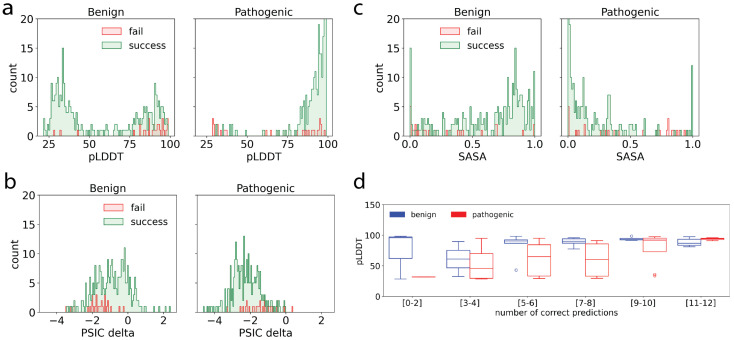
Feature distributions and HerCanPred_*rf*_ prediction outcomes. Distribution of successful (green) and failed (red) predictions of HerCanPred_*rf*_ for benign and pathogenic variants (*n* = 309 for each) based on three most important features: (**a**) pLDDT, (**b**) ΔPSIC scores, and (**c**) relative accessible surface area (SASA). (**d**) Distribution of the number of correct predictions among 12 different predictors other than HerCanPred_*rf*_.

We further analyzed the distributions of the three most important features—pLDDT, rASA and ΔPSIC—for the successful and unsuccessful predictions for pathogenic and benign variants (Figure 5). For benign variants, failed predictions were spread across both low and high pLDDT values, indicating challenges in predicting across a large pLDDT range (Figure 5a). In contrast, for pathogenic variants, failed predictions were predominantly found in regions with low pLDDT scores, suggesting that pathogenic mutations in structurally less confident or disordered regions are more difficult to classify accurately by HerCanPred_*rf*_. ΔPSIC distributions showed that both benign and pathogenic variants had a similar trend where both successful and failed predictions cluster around negative ΔPSIC values (Figure 5b). This indicates that mutations in moderately or weakly conserved positions tend to be more challenging for the predictor, particularly in pathogenic variants. SASA distributions elucidated that in benign variants, failed predictions were spread across a broad range of SASA values (Figure 5c). On the other hand, for pathogenic variants, failed predictions were concentrated at high SASA values, indicating that surface-exposed residues might be more challenging to classify accurately, possibly due to their dynamic nature or functional complexity. The clustering of failed predictions in low-pLDDT and high-SASA regions for pathogenic variants suggests that mutations in structurally uncertain or disordered regions pose a greater challenge for pathogenicity classification. These observations not only highlight the importance of structural features but also suggest that additional contextual information, such as interaction data, for the surface-exposed and low-pLDDT variants could improve their prediction accuracy.

Figure 5d shows distributions of pLDDT scores in pathogenic and benign variants stratified by the number of predictors that produced a correct result. Benign and pathogenic variants located in high-confidence regions (high pLDDT scores) are enriched in the [9–10] and [11–12] correct prediction groups. This clustering indicates improved classification consistency for variants with high pLDDT values. On the other hand, variants with low pLDDT scores were predominantly misclassified by multiple predictors. Overall, these results suggest that predictors perform more reliably when structural confidence is high, particularly for pathogenic variants. The consistent misclassification of low-pLDDT variants underscores the importance of incorporating structural confidence metrics into prediction models. Enhancing model performance in these regions may require integrating additional data, such as protein dynamics or interaction context.

One notable point was that pathogenic variants tended to show more variable pLDDT distributions, particularly for the [5–6], [7–8] and [9–10] correct prediction groups (Figure 5d), indicating that pLDDT score is a stronger determinant of prediction success for pathogenic variants than for benign variants.

### 2.6. Reclassification of VUS Variants by Top Classifiers

**Figure 6:**
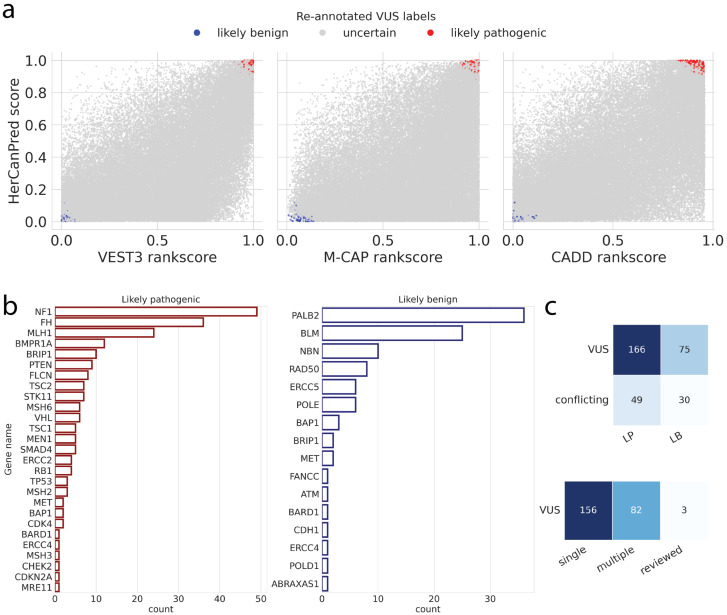
Reclassification of ClinVar VUS (*n* = 57,662) (**a**) shows joint distributions between HerCanPred scores (y-axis) and three classifiers, VEST3, M-CAP, and CADD. Variants with mean scores above 0.95 were re-annotated as likely pathogenic (*n* = 215) (red), and those below 0.05 as likely benign (*n* = 105) (blue). (**b**) Distribution of reclassified VUS variants, likely pathogenic variants (red) and likely benign variants (blue), in genes. (**c**) Distribution of reclassified variants with respect to (**top**) ClinVar labels of VUS or conflicting interpretations and their reassignment as likely pathogenic (LP) or likely benign (LB) and (**bottom**) ClinVar review status: single submitter, multiple submitters or expert-reviewed.

To refine the interpretation of VUS, we applied a re-annotation strategy based on distributions of existing variant classifiers and HerCanPred scores, followed by gene-level assessments and comparison to ClinVar review categories. Figure 6a shows the joint score distributions of HerCanPred with three independent variant effect predictors (VEST3, M-CAP, and CADD). While the majority of VUS remained in the intermediate range (gray), variants with consistently high (>0.95) or low (<0.05) mean classifier scores were re-annotated as likely pathogenic (red) or likely benign (blue), respectively. This approach enabled the separation of a small but distinct subset of variants from the large background of uncertain cases. The concordance of classifier scores at the extremes provided increased confidence for reclassification.

We next examined the gene-level distribution of variants that were reassigned to likely pathogenic or likely benign categories (Figure 6b). Genes with the highest numbers of reclassified likely pathogenic variants included *NF1*, *FH*, and *MLH1*, reflecting their established roles in cancer predisposition. Additional recurrently affected genes included *BMPR1A*, *BRIP1*, *PTEN*, *FLCN*, and *STK11*. In contrast, the largest contributors to the reclassified likely benign set were *PALB2*, *BLM*, and *NBN*, followed by *RAD50* and *ERCC5*. These results suggest that while certain tumor suppressors and cancer-associated genes harbor many reclassified likely pathogenic variants, other DNA-repair-related genes are enriched in variants reclassified as likely benign.

We further assessed how these reclassified variants were distributed across existing ClinVar categories (Figure 6c). The majority originated from entries previously labeled as VUS (166 reclassified as likely pathogenic and 75 as likely benign), with a smaller subset from conflicting interpretations (49 to likely pathogenic and 30 to likely benign). When stratified by ClinVar review status, most reclassified variants were derived from submissions with single-submitter evidence (156), followed by multiple submitters without consensus (82), with very few from expert-reviewed entries (3). This pattern highlights that the largest potential for reclassification lies in variants with lower review confidence, while variants with expert consensus remain largely stable.

Together, these analyses show that combining orthogonal variant effect predictors with HerCanPred scores could contribute to reclassification efforts for VUS variants. The gene-level patterns reinforce the biological plausibility of these reclassifications, with well-established cancer genes enriched for likely pathogenic variants. Finally, comparison to ClinVar categories reveals that most reclassifications affect variants from lower-confidence annotation tiers, such as those with single submitters, providing a rational basis for updating their clinical interpretation.

## 3. Discussion

To address the growing clinical need for reliable tools to interpret missense variants in hereditary cancer, we developed HerCanPred, a targeted pathogenicity predictor for 63 cancer-predisposition genes. HerCanPred’s performance advantage is evident when compared against established variant effect predictors on hereditary cancer genes. While general algorithms such as SIFT and PolyPhen-2 were originally developed for broad application across the genome, their predictive power appears limited in the context of our cancer gene panel. In our evaluations, HerCanPred produced consistently superior classification performance, reflected in both AUROC and precision–recall metrics. For instance, the HerCanPred_*rf*_ model maintained better F1 and MCC scores than those of SIFT or PolyPhen-2 on the same variant set. Even ensemble methods like MetaSVM, which integrate multiple predictors, lag behind. These results indicate that a focused tool trained on disease-specific data can achieve both higher sensitivity and specificity, an observation consistent with prior reports that accuracy varies by gene and that tailored or ensemble approaches can outperform single generic tools [24]. It is notable that HerCanPred also slightly outperformed VEST3, suggesting a meaningful advance over known methods.

Beyond predictive performance on known pathogenic/benign variants, a critical outcome of this study is the reclassification of VUS. VUS constitute a large fraction of findings in clinical genetic testing. In a recent 1.7 million-person cohort, roughly 41% of individuals had at least one VUS identified (mostly missense changes) [17]. Such high prevalence of uncertain results underscores the pressing need for improved methods to evaluate missense variants and reduce diagnostic uncertainty [17]. Our analysis showed that HerCanPred, especially when used in conjunction with orthogonal predictors, can help resolve a subset of these ambiguities. By applying mean classifier score thresholds, we were able to reclassify hundreds of previously unclassified variants as likely pathogenic or likely benign. Specifically, 166 variants initially labeled as VUS were reassigned as likely pathogenic and 75 as likely benign, with additional reclassifications from the “conflicting interpretation” category. Importantly, the vast majority of these reclassified variants originated from lower-confidence ClinVar entries, highlighting that the greatest potential for reclassification lies in variants lacking strong prior evidence, aligning with expert recommendation that variant interpretations should be periodically revisited as new data emerge. Indeed, there is growing recognition in the clinical genomics community that systematic variant reinterpretation is necessary for patient care, prompting calls for clearer guidelines and scalable approaches to reevaluate VUS over time [20]. Our results contribute to this effort by providing a computational tool that can flag likely benign or pathogenic cases among thousands of VUS, thereby offering a rational basis to update their clinical classification. Gene-level reclassification patterns provide additional biological support for these updates. For example, newly pathogenic classifications are enriched in *NF1*, *FH*, and *MLH1*, whereas reclassifications in *PALB2* and *BLM* are predominantly benign. Overall, the ability of HerCanPred to facilitate VUS reclassification addresses a significant clinical need: it can help reduce the burden of uncertainty in hereditary cancer testing by converting uncertain results into more definitive interpretations, ultimately guiding more informed medical management [17]. Specifically, this tool supports the prioritization and interpretation of germline VUS in hereditary cancer testing by enabling the reporting of clinically prioritized or potentially harmful variants to clinicians, thereby informing genetic counseling for patients and their families, facilitating disease association assessments, supporting cascade screening of relatives at risk, and helping to ensure timely early diagnosis, personalized surveillance, and appropriate long-term clinical follow-up. The current model is restricted to germline missense variants in a predefined hereditary cancer gene panel and does not account for zygosity, somatic variation, or non-missense variant classes, which limits direct generalizability beyond the studied scope.

HerCanPred’s strong performance can be attributed in part to its comprehensive feature set, which integrates both sequence-based and structure-based information for each variant. The model leverages evolutionary conservation metrics alongside protein structural features derived from in silico models. SHAP analysis identified two AF2-derived structural features as the strongest drivers of pathogenicity predictions: the local pLDDT confidence score and the rASA of the substituted residue. Lower pLDDT and higher rASA were strongly associated with pathogenic variants. This finding aligns with emerging evidence in the literature that incorporating protein structural context can enhance missense variant classifiers. For example, a recent study introduced an AlphaFold-based score (AlphScore) using features like solvent accessibility and residue interaction networks, and showed that adding such structure-derived features improved prediction of ClinVar pathogenic variants [21,25]. At a broader level, several recent approaches have incorporated structural and biophysical features to improve predictive performance, ranging from ensemble methods that integrate protein stability metrics to deep learning models that explicitly leverage the three-dimensional protein context [26]. Our work reinforces this paradigm: HerCanPred’s integration of sequence conservation with 3D structural insights enabled it to detect subtle perturbations that distinguish pathogenic variations (such as those destabilizing a protein fold or occurring in highly constrained regions). By leveraging the AF2 structural proteome [21,25], we also overcame the limitation of sparse experimental structures and extracted new predictors of variant impact. Overall, our results show that combining evolutionary evidence with structure-based features improves both the accuracy and generalizability of variant effect classifiers for hereditary cancer genes. Integrating protein dynamics or interaction data could help resolve challenges in these structurally uncertain regions [27,28,29].

While pLDDT is fundamentally a structural confidence metric, recent studies have demonstrated that pLDDT scores are also effective indicators of intrinsic disorder and structural flexibility [21,30,31,32]. Low pLDDT values strongly correlate with disordered regions, making pLDDT a useful proxy for both prediction confidence and structural disorder. From this perspective, our observations align with these established findings that structural disorder is enriched in neutral variants but depleted among pathogenic variants [30,33]. Pathogenic variants predominantly occur in structurally ordered regions with well-defined conformations, whereas neutral variants are more frequently tolerated in disordered or flexible regions. Therefore, the higher pLDDT scores and lower rASA values observed for pathogenic variants reflect their characteristic localization in functionally constrained, well-ordered regions where mutations are more likely to disrupt critical structural or functional properties. Importantly, the use of AF2 structures provided advantages over experimental structures by ensuring comprehensive coverage of all protein regions, including those with structural uncertainty (low pLDDT), thereby reducing bias and improving the completeness of our variant pathogenicity predictions [21].

To isolate the drivers of pathogenicity, we compared the full HerCanPred_*rf*_ model against a reduced version (HerCanPred_*rf*__*Red.*_) excluding gene-level features. This ablation analysis revealed that variant-specific sequence and structural properties capture the vast majority of the pathogenic signal (Table 2). On the other hand, although moderate, the performance gain from gene-level features also aligns with the prior findings establishing a relationship between gene-level constraints and pathogenicity [34]. Overall, this analysis further supported the biological validity of our feature set while confirming that the model’s core performance stems from variant-level characteristics.

Several limitations should also be considered when interpreting our results. First, HerCanPred was trained exclusively on ClinVar-derived labels for missense variants in 63 hereditary cancer genes and thus remains sensitive to residual misclassification and uneven curation across genes; moreover, its applicability outside this gene set and in non-coding or truncating variants has not been evaluated. Second, the model relies on in silico features, including AF2-derived structural annotations, which may be less reliable in intrinsically disordered regions or for proteins with substantial conformational flexibility. Third, our VUS reclassification analysis is based on computational evidence and retrospective ClinVar data only, without prospective clinical follow-up. To address these limitations, future work should prioritize functional validation of computationally reclassified variants using gene- and domain-specific assays such as saturation mutagenesis, multiplexed reporter assays, DNA repair and signaling readouts in appropriate cell models, or CRISPR-based perturbation in patient-derived organoids. Integrating such experimental measurements with longitudinal clinical data and co-segregation evidence will be essential in refining HerCanPred’s decision thresholds, recalibrating its predictions across diverse populations, and formally incorporating its output as supporting or moderate evidence within existing ACMG/AMP frameworks. Together, these extensions would strengthen the clinical validity of HerCanPred and enable its responsible deployment as a decision-support tool for hereditary cancer variant interpretation.

## 4. Materials and Methods

### 4.1. Hereditary Cancer Gene Panels

In this study, two widely utilized hereditary cancer gene panels were used, consisting of 26 and 59 genes [35,36]. The union of these panels resulted in a non-redundant set of 63 unique genes. Additionally, all cancer-related genes reported in Online Mendelian Inheritance in Man (OMIM), The Human Phenotype Ontology (HPO), and ClinVar were collected [37,38,39], compiling a larger gene set of 902 genes (see Supplementary Materials). Protein–protein interactions for this gene set were analyzed using the STRING database v10.5 [40] and Cytoscape v3.10.4 [41].

### 4.2. Dataset Collection

A comprehensive dataset of missense variants for the 63 hereditary cancer-associated genes was compiled from the ClinVar database [19], queried on 28 June 2023. Missense variants with classifications (pathogenic, likely pathogenic, benign, likely benign, VUS or conflicting interpretations), germline origin, and review status ≥ 1 star were filtered. Variants with somatic origin were excluded. The ClinVar query details are shared in the Supplementary Materials. Gene symbols were mapped to UniProt canonical (longest) isoforms, as they correspond to structures in the AlphaFold DB (v4). Variants with mapping inconsistencies between ClinVar annotations and UniProt sequences were removed. AF2 structures were retrieved for all proteins, providing full-length structural predictions with complete residue coverage. The mapping pipeline yielded 60,753 variants across 63 hereditary cancer genes.

### 4.3. Feature Extraction

A diverse set of predictive features was computed for each variant. Features included Gene Ontology (GO) term counts, position-specific independent counts (PSIC) [42,43,44], physicochemical properties (hydrophobicity, volume), secondary structure propensities, amino acid frequencies, and BLOSUM62 substitution scores [45]. These features were complemented by structural features, namely, the per-residue pLDDT score and solvent accessible surface area (SASA), which were extracted from protein structures predicted by AF2 in the AlphaFold DB (v4) [46,47]. Relative surface accessible surface area (rASA), which is a normalized measure of how exposed an amino acid residue is to a solvent, is calculated by dividing the observed solvent accessible surface area (SASA) of a residue by the maximum possible SASA for that amino acid type [48] via the DSSP module of BioPhyton.PDB v1.86 [49]. The dataset is available at https://github.com/timucinlab/HerCanPred (accessed on 12 February 2026).

### 4.4. Model Development

For binary classification of variants as pathogenic versus benign, two algorithms, random forest and extreme gradient boosting (XGBoost v3.2.0), were developed [50]. The data was first partitioned into a training and a held-out test set, including 2473 and 618 variants, respectively. Model development used nested cross-validation exclusively on the training set, with a 5-fold outer loop to estimate the performance and a 3-fold inner loop for hyperparameter tuning. Hyperparameters were optimized via Bayesian optimization with Hyperopt [51], running 150 trials within each inner loop. SHapley Additive exPlanations (SHAP) analysis was conducted for model interpretability [52]. SHAP computations were performed within the training folds of the cross-validation procedure. After model selection, the model was refitted on the full training set and evaluated once on the held-out test set for the final performance estimate.

To evaluate the contribution of gene-level and annotation-density features such as GO term counts and protein length versus variant-specific features, we trained two versions of the random forest classifier including (i) all features and (ii) a reduced model excluding gene/protein-level features while only retaining sequence- and structure-based variant features. Both models were trained and evaluated using identical cross-validation procedures on the same dataset.

### 4.5. Performance Evaluation and VUS Reclassification

The predictive performance of the trained models was benchmarked against established classifiers. Twenty-three tools were selected based on their availability in ANNOVAR v2025Jul29, a widely used variant annotation framework providing standardized access to pre-computed pathogenicity predictions. It integrates diverse prediction algorithms including conservation-based methods (GERP++, phyloP, phastCons, SiPhy), functional predictors (SIFT, PolyPhen-2, PROVEAN, MutationTaster, MutationAssessor, FATHMM), and ensemble methods (MetaSVM, MetaLR, M-CAP, CADD, VEST3), representing the most commonly benchmarked tools through ANNOVAR [53]. The evaluation was based on a set of metrics, including the area under the receiver operating characteristic curve (AUROC), F1 score, Matthews correlation coefficient (MCC), and precision. We further employed bootstrap resampling to calculate confidence intervals and assess statistical significance of performance differences. For each method, 1000 bootstrap samples were generated by random sampling of the hold-out test set with replacement, maintaining the original sample size. Bootstrap samples that did not contain both classes (pathogenic and benign) were excluded from analysis. Finally, the best-performing methods according AUROC-based performance were used to predict 57,662 VUS and conflicting interpretations. These reclassifications are available at https://github.com/timucinlab/HerCanPred.

## 5. Conclusions

In conclusion, this study introduces HerCanPred, a disease-focused pathogenicity prediction framework that utilizes evolutionary, physicochemical, and AF2-derived structural features to improve the interpretation of missense variants in hereditary cancer genes. By outperforming widely used generic predictors and enabling systematic reclassification of a substantial subset of VUS, HerCanPred addresses a critical bottleneck in clinical cancer genetics—the high burden of uncertain variant interpretations. Its ability to integrate interpretable structural signals and gene-specific patterns provides a rational and scalable approach for variant prioritization, supporting more consistent and clinically meaningful decision-making. While further functional validation and prospective clinical integration are required, HerCanPred represents a significant step toward reducing diagnostic uncertainty and advancing precision medicine in hereditary cancer risk assessment.

## Figures and Tables

**Table 1:** Nested cross-validation results.

	AUROC	F1	Precision	Recall	MCC	Accuracy
HerCanPred_*rf*_	0.95 ± 0.01	0.89 ± 0.01	0.89 ± 0.01	0.89 ± 0.02	0.78 ± 0.03	0.89 ± 0.01
HerCanPred_*xgb*_	0.94 ± 0.02	0.88 ± 0.03	0.87 ± 0.04	0.88 ± 0.03	0.75 ± 0.06	0.88 ± 0.03

**Table 2:** Classification performance metrics.

Predictor *	Size	AUROC	F1	MCC
HerCanPred_*xgb*_	618	0.951	0.896	0.792
HerCanPred_*rf*___*Red.*_	618	0.930	0.862	0.735
HerCanPred_*rf*_	618	0.963 [0.948, 0.976]	0.854 [0.820, 0.885]	0.716 [0.659, 0.773]
SIFT	612	0.881 [0.853, 0.907]	0.813 [0.778, 0.843]	0.597 [0.540, 0.655]
Polyphen2_HDIV	617	0.862 [0.831, 0.889]	0.814 [0.781, 0.844]	0.600 [0.540, 0.655]
Polyphen2_HVAR	617	0.875 [0.845, 0.899]	0.822 [0.789, 0.852]	0.623 [0.562, 0.681]
LRT	603	0.850 [0.819, 0.881]	0.822 [0.790, 0.851]	0.616 [0.558, 0.675]
MutationTaster	617	0.818 [0.786, 0.849]	0.790 [0.758, 0.822]	0.545 [0.492, 0.597]
MutationAssessor	596	0.873 [0.846, 0.898]	0.802 [0.770, 0.833]	0.581 [0.519, 0.638]
FATHMM	615	0.738 [0.698, 0.777]	0.687 [0.646, 0.726]	0.297 [0.224, 0.369]
PROVEAN	614	0.887 [0.862, 0.911]	0.795 [0.760, 0.829]	0.585 [0.518, 0.649]
MetaSVM	617	0.850 [0.815, 0.879]	0.794 [0.756, 0.828]	0.576 [0.507, 0.640]
MetaLR	617	0.834 [0.799, 0.863]	0.763 [0.725, 0.798]	0.493 [0.425, 0.559]
M-CAP	553	0.899 [0.874, 0.922]	0.744 [0.709, 0.778]	0.374 [0.328, 0.421]
fathmm-MKL	617	0.843 [0.811, 0.874]	0.785 [0.752, 0.817]	0.533 [0.481, 0.590]
VEST3	615	0.932 [0.914, 0.950]		
CADD_raw	617	0.901 [0.874, 0.924]		
DANN	617	0.803 [0.770, 0.838]		
GenoCanyon	617	0.703 [0.663, 0.741]		
integrated_fitCons	617	0.574 [0.527, 0.622]		
GERP++_RS	617	0.826 [0.792, 0.859]		
phyloP100way	617	0.844 [0.812, 0.875]		
phyloP20way	617	0.731 [0.689, 0.772]		
phastCons100way	617	0.785 [0.753, 0.817]		
phastCons20way	617	0.780 [0.748, 0.815]		
SiPhy_29way	617	0.825 [0.791, 0.857]		

* Predictors without a classification outcome were evaluated based on AUROC. We calculated 95% confidence intervals using 1000 bootstrap iterations.

## Data Availability

The dataset, models and VUS reclassifications are available at https://github.com/timucinlab/HerCanPred.

## Supplementary Materials

The following supporting information can be downloaded at: https://www.mdpi.com/article/10.3390/ijms27052453/s1.
