# Supplementary material for: Pathogenicity Prediction of Missense Variations in Hereditary Cancer Genes
Source: Int J Mol Sci. 2026 Mar 7;27(5):2453. doi: 10.3390/ijms27052453 (PMC12985416; doi:10.3390/ijms27052453)
Supplement: Supplementary file 1 [file ijms-27-02453-s001.zip › ijms-4132908-supplementary.pdf]

Supplementary Information

1. ClinVar Query:

```
(APC[Gene Name]) OR (ATM[Gene Name]) OR (AXIN2[Gene Name]) OR
(BAP1[Gene Name]) OR (BARD1[Gene Name]) OR (BLM[Gene Name]) OR
(BMPR1A[Gene Name]) OR (BRCA1[Gene Name]) OR (BRCA2[Gene Name]) OR
(BRIP1[Gene Name]) OR (CDH1[Gene Name]) OR (CDK4[Gene Name]) OR
(CDKN2A[Gene Name]) OR (CHEK2[Gene Name]) OR (DDB2[Gene Name]) OR
(EPCAM[Gene Name]) OR (ERCC2[Gene Name]) OR (ERCC3[Gene Name]) OR
(ERCC4[Gene Name]) OR (ERCC5[Gene Name]) OR (FANCA[Gene Name]) OR
(FANCC[Gene Name]) OR (FH[Gene Name]) OR (FLCN[Gene Name]) OR
(GALNT12[Gene Name]) OR (HDAC2[Gene Name]) OR (HOXB13[Gene Name]) OR
(MEN1[Gene Name]) OR (MET[Gene Name]) OR (MITF[Gene Name]) OR
(MLH1[Gene Name]) OR (MSH2[Gene Name]) OR (MSH3[Gene Name]) OR
(MSH6[Gene Name]) OR (MUTYH[Gene Name]) OR (NBN[Gene Name]) OR
(NF1[Gene Name]) OR (NF2[Gene Name]) OR (NTHL1[Gene Name]) OR
(PALB2[Gene Name]) OR (PMS2[Gene Name]) OR (POLD1[Gene Name]) OR
(POLE[Gene Name]) OR (POLH[Gene Name]) OR (PTCH1[Gene Name]) OR
(PTEN[Gene Name]) OR (RAD51C[Gene Name]) OR (RAD51D[Gene Name]) OR
(RB1[Gene Name]) OR (RET[Gene Name]) OR (SMAD4[Gene Name]) OR
(STK11[Gene Name]) OR (TP53[Gene Name]) OR (TSC1[Gene Name]) OR
(TSC2[Gene Name]) OR (VHL[Gene Name]) OR (WT1[Gene Name]) OR
(XPA[Gene Name]) OR (XPC[Gene Name])
```

2. Panel Genes

26 genes: ABRAXAS1, ATM, BARD1, BLM, BRCA1, BRCA2, BRIP1, CDH1, CHEK2, EPCAM, MEN1, MLH1, MRE11, MSH2, MSH6, MUTYH, NBN, PALB2, PMS2, PTEN, RAD50, RAD51C, RAD51D, STK11, TP53 ve XRCC2

59 genes: APC, ATM, AXIN2, BAP1, BARD1, BLM, BMPR1A, BRCA1, BRCA2, BRIP1, CDH1, CDK4, CDKN2A, CHEK2, DDB2, EPCAM, ERCC2, ERCC3, ERCC4, ERCC5, FANCA, FANCC, FH, FLCN, GALNT12, HDAC2, HOXB13, MEN1, MET, MITF, MLH1, MSH2, MSH3, MSH6, MUTYH, NBN, NF1, NF2, NTHL1, PALB2, PMS2, POLD1, POLE, POLH, PTCH1, PTEN, RAD51C, RAD51D, RB1, RET, SMAD4, STK11, TP53, TSC1, TSC2, VHL, WT1, XPA, XPC

319 genes: ABCA3, ABCB7, ABCG5, ABCG8, ACTB, ACTN1, ADAMTS13, AIP, AK1, AK2, ALAS2, ALK, AMN, ANK1, ANKRD26, AP3B1, AP3D1, APC, ATM, ATR, ATRX, AXIN2, BAP1, BARD1, BLM, BLOC1S3, BLOC1S6, BMPR1A, BRAF, BRCA1, BRCA2, BRIP1, BUB1B, CBL, CD59, CDAN1, CDC42, CDC73, CDH1, CDK4, CDKN1B, CDKN1C, CDKN2A, CEBPA, CEP57, CHEK2, CLCN7, CLPB, CSF2RA, CSF3R, CTC1, CTLA4, CTNNA1, CTSC, CUBN, CXCR4, CYB5R3, CYLD, DDB2, DHFR, DICER1, DIS3L2, DKC1, DNASE2, DTNBP1, EGFR, EGLN1, ELANE, EPAS1, EPB41, EPB42, EPCAM, EPOR, ERCC1, ERCC2, ERCC3, ERCC4, ERCC5, ETV6, EXO1, EXT1, EXT2, EZH2, F10, F11, F12, F13A1, F13B, F2, F5, F7, F8, F9, FADD, FANCA, FANCB, FANCC, FANCD2, FANCE, FANCF, FANCG, FANCI, FANCL, FANCM, FAS, FASLG, FGA, FGB, FGG, FH, FLCN, FLI1, FLNA, G6PC3, G6PD, GALNT12, GATA1, GATA2, GBA, GCLC, GFI1, GFI1B, GGCX, GLRX5, GP1BA, GP1BB, GP9, GPC3, GPI, GPR143, GREM1, GSS, HAX1, HBB, HFE, HK1, HMOX1, HNF1A, HOXA11, HOXB13, HPS1, HPS3, HPS4, HPS5, HPS6, HRAS, IFNGR2, IKZF1, ITGA2, ITGA2B, ITGB3, ITK, JAK2, KIF1B, KIT, KITLG, KLF1, KRAS, LAMTOR2, LMAN1, LPIN2, LYST, LZTR1, MAGT1, MAP2K1, MAP2K2, MASTL, MAX, MCFD2, MECOM, MEN1, MET, MITF, MLH1, MLH3, MPL, MSH2, MSH3, MSH6, MTHFD1, MTR, MUTYH, MYH9,

MYO5A, NBEAL2, NBN, NF1, NF2, NOP10, NRAS, NSD1, NSUN2, NT5C3A, NTHL1, OCA2, P2RY12, PALB2, PARN, PAX5, PC, PDGFRA, PDHA1, PDHX, PGK1, PHOX2B, PKLR, PMS1, PMS2, POLD1, POLH, POT1, PPM1D, PRF1, PRKAR1A, PROC, PROS1, PTCH1, PTEN, PTPN11, PUS1, RAB27A, RAC2, RAD50, RAD51C, RAD51D, RAF1, RASGRP2, RB1, RECQL4, REN, RET, RHAG, RHBDF2, RIT1, RNF168, RPL11, RPL35A, RPL5, RPS19, RPS24, RPS26, RPS7, RRAS, RTEL1, RUNX1, SAMD9, SBDS, SDHA, SDHAF2, SDHB, SDHC, SDHD, SEC23B, SERPINC1, SERPINF2, SFTPB, SFTPC, SH2D1A, SHOC2, SLC11A2, SLC19A2, SLC25A38, SLC37A4, SLC45A2, SLC46A1, SLC4A1, SLX4, SMAD4, SMARCA4, SMARCB1, SMARCE1, SOS1, SOS2, SPRED1, SPTA1,SPTB,SRC, SRP72, STAT3, STK11, STX11, STXBP2, SUFU, TBXA2R, TBXAS1, TCN2, TERC, TERT, TF, THBD, THPO, TINF2, TMEM127, TMPRSS6, TP53, TRIP13, TRNT1, TSC1, TSC2, TUBB1, TYR, TYRP1, UNC13D, USB1, VHL, VKORC1, VPS13B, VWF, WAS, WIPF1, WRAP53, WRN, WT1, XIAP, XPA, XPC, XRCC2, YARS2, ZCCHC8

769 genes: AAGAB, ABCB11, ABCC6, ABCC8, ABL1, ABL2, ABRAXAS1, ACAN, ACP5, ACTB, ACVR1, ACVR2A, ACVRL1, ADA, ADAR, AFF3, AHCY, AIP, AKAP9, AKT1, AKT2, AKT3, ALK, ALX1, ALX3, ALX4, ANTXR1, ANTXR2, APC, AR, ARHGAP26, ARID1A, ARID1B, ARSA, ASCC1, ASCL1, ASXL1, ATM, ATP7A, ATP7B, ATR, ATRX, AURKA, AURKC, AXIN1, AXIN2, AXL, B3GALT6, BAP1, BARD1, BAX, BCHE, BCL2, BCL2L1, BCL2L2, BCL6, BCL9, BCL10, BCL11A, BCR, BICC1, BIN1, BLK, BLM, BLNK, BMP2, BMPER, BMPR1A, BMPR1B, BRAF, BRCA1, BRCA2, BRIP1, BTK, BUB1, BUB1B, C1S, CACNA1S, CALR, CARD11, CARD14, CASP8, CASP10, CASR, CAT, CBFB, CBL, CC2D2A, CCBE1, CCDC22, CCL2, CCM2, CCND1, CD19, CD27, CD79A, CD79B, CD81, CD96, CDC73, CDH1, CDH5, CDH23, CDK4, CDK6, CDKN1B, CDKN1C, CDKN2A, CDKN2B, CDKN2C, CDON, CEBPA, CEL, CEP57, CHD7, CHEK2, CHIC2, CHRNG, CIC, CLCNKB, CMPK1, COL1A1, COL2A1, COL4A5, COL7A1, COL11A2, COL18A1, COMP, CPLANE1, CPOX, CR2, CRBN, CREB1, CREBBP, CRKL, CSF1R, CSMD3, CTC1, CTHRC1, CTLA4, CTNNB1, CTSA, CTSC, CXCR4, CYLD, CYP2A6, CYP2C19, CYP2D6, CYP11B1, CYP11B2, CYP26C1, DCC, DCLRE1C, DDB2, DDR2, DHCR7, DHCR24, DHH, DHX37, DICER1, DIS3L2, DKC1, DLC1, DLL1, DMPK, DNASE1L3, DNM2, DNMT3A, DOCK8, DPM1, DPYD, DST, DYNC2H1, ECE1, ECM1, EDN1, EDN3, EDNRB, EGFR, ELANE, ENG, ENPP1, EP300, EPAS1, EPCAM, EPHA3, EPHA7, EPHB2, EPHB6, ERBB2, ERBB3, ERBB4, ERCC1, ERCC2, ERCC3, ERCC4, ERCC5, ERCC6, ESCO2, ESR1, ETV4, ETV6, EVC, EVC2, EWSR1, EXT1, EXT2, EXTL3, EYA1, EZH2, F5, F13A1, F13B, FAH, FAM20C, FAN1, FANCA, FANCB, FANCC, FANCD2, FANCE, FANCF, FANCG, FANCI, FANCL, FANCM, FAS, FASLG, FBXW7, FCN3, FERMT1, FGF3, FGF8, FGFR1, FGFR2, FGFR3, FGFR4, FGFRL1, FH, FLCN, FLI1, FLNA, FLT1, FLT3, FLT4, FN1, FOXC2, FOXE1, FOXI1, FOXL2, FOXO1, FOXP1, FUZ, G6PC, G6PD, GABRD, GALNT12, GATA1, GATA2, GATA3, GATA4, GBA, GCDH, GCGR, GCK, GCM2, GDF5, GDNF, GFI1, GFI1B, GJB2, GJB3, GJB4, GJB6, GJC2, GLI2, GLI3, GNAI3, GNAQ, GNAS, GNPTAB, GPC3, GPC4, GPC6, GPR143, GREM1, GTF2H5, HABP2, HACE1, HAX1, HBB, HFE, HIF1A, HMBS, HMGA2, HMMR, HNF1A, HNF4A, HOXB13, HOXD13, HPGD, HRAS, HSP90AA1, HSPA9, HSPG2, ICOS, IDH1, IDH2, IFIH1, IFNG, IGF1R, IGF2, IGF2R, IGLL1, IKBKB, IKZF1, IL1B, IL1RN, IL2RG, IL6, IL6ST, IL7R, IL12RB1, IL21R, ING1, INPP5E, INS, IRF1, IRF4, IRF5, IRS2, ITGA9, ITGB2, ITGB3, ITK, JAG1, JAK2, JAK3, JUN, KAT6A, KAT6B, KCNE3, KCNJ10, KCNJ11, KCNN3, KCNQ1, KDM5C, KDM6A, KDM6B, KDR, KIF1B, KIF7, KIF11, KIT, KLF6, KLF11, KLHDC8B, KRAS, KRT1, KRT5, KRT6A, KRT9, KRT10, KRT14, KRT16, KRT17, L2HGDH, LAMA3, LAMB3, LAMC2, LCK, LEMD3, LETM1, LIFR, LIG4, LMNA, LMX1B, LPP, LRP1B, LRP5, LRRC8A, LYST, LZTS1, MAD1L1, MAF, MAFA, MAFB, MAGT1, MALT1, MAML2, MAP2K1, MAP2K2, MAP2K4, MAP3K1, MAP3K8, MAPK1, MAX, MBD1, MBTPS2, MC1R, MC2R, MCC, MCM4, MDM2, MEN1, MET, MFN2, MGAT2, MINPP1, MITF, MLH1, MLH3, MLLT10, MMP1, MMP2, MN1, MNX1, MPL, MPLKIP, MRAP, MRE11, MS4A1, MSH2, MSH3, MSH6, MSR1, MST1R, MSX2, MTAP, MTM1, MTMR14, MTR, MTRR, MUC5B, MUTYH, MVK, MXI1, MYB, MYC, MYCN, MYD88, MYH8, MYH9, MYH11, MYLK, NAGS, NBEAL2, NBN,, NCOA1, NDP, NDUFAF6, NEK1, NEUROD1, NF1, NF2, NFKB2, NIN, NKX2-1, NLRP1, NNT, NOD2, NODAL, NOP10, NOTCH1, NOTCH2, NOTCH3, NPM1, NR4A3, NR5A1, NRAS, NR0B1, NSD1, NSD2, NSUN2, NTHL1, NTRK1, NTRK3, NUMA1, NUP214, OCRL, ODC1, OFD1, OGG1, OPCML, PAK3, PALB2,

PALLD, PARP1, PAX3, PAX4, PAX5, PAX6, PAX7, PAX8, PBX1, PDCD10, PDGFB, PDGFRA, PDGFRB, PDGFRL, PDX1, PER1, PHB, PHKA2, PHKG2, PHOX2B, PICALM, PIGA, PIGL, PIK3CA, PIK3CD, PIK3R1, PIK3R2, PIM1, PKD1, PKD2, PKHD1, PLA2G2A, PLAG1, PLCB4, PLCD1, PLEKHG5, PML, PMS1, PMS2, PNP, POLD1, POLH, POLR1C, POLR1D, PORCN, POU5F1, POU6F2, PPARG, PPM1D, PPOX, PPP2R1B, PRCC, PRDM16, PRF1, PRKAR1A, PRKDC, PRKN, PRLR, PRSS1, PSAP, PSENEN, PTCH1, PTCH2, PTEN, PTGS2, PTH1R, PTPN11, PTPN12, PTPRJ, PTPRT, PYGL, RAD21, RAD50, RAD51, RAD51C, RAD51D, RAD54B, RAD54L, RAF1, RAG1, RAG2, RALGDS, RARA, RASA1, RASGRP1, RB1, RB1CC1, RECQL4, REL, RET, RHBDF2, RHOH, RNASEH2A, RNASEH2B, RNASEH2C, RNASEL, RNF6, RNF113A, RNF139, RNF213, ROS1, RPGRIP1L, RPL5, RPL10, RPL11, RPL35A, RPS19, RPS24, RPS26, RRAS2, RSPO1, RYR1, SAMD9, SAMHD1, SCN4A, SCN9A, SCN10A, SCN11A, SDHA, SDHAF2, SDHB, SDHC, SDHD, SEC23A, SEC23B, SEMA4A, SETBP1, SETD2, SF3B1, SFTPA2, SFTPC, SH2B3, SH2D1A, SH3GL1, SHH, SHOX, SIX1, SIX3, SIX6, SKI, SLC12A3, SLC22A18, SLC25A13, SLC26A2, SLC26A4, SLC37A4, SLC45A2, SLCO2A1, SLX4, SMAD2, SMAD4, SMAD7, SMARCA2, SMARCA4, SMARCAD1, SMARCB1, SMARCE1, SMO, SMPD1, SMUG1, SNAI2, SOS1, SOX2, SOX6, SOX9, SPINK1, SPRED1, SQSTM1, SRC, SRD5A3, SRP72, SRY, STAR, STAT1, STAT3, STIM1, STK4, STK11, STK36, STS, SUFU, SYK, SYNE1, TAF1, TAF1L, TAL1, TAL2, TBC1D24, TBX2, TBX22, TBXT, TCF3, TCF4, TCF7L1, TCF7L2, TCIRG1, TCOF1, TCTN3, TEK, TERT, TET1, TET2, TFAP2A, TFE3, TG, TGFBR1, TGFBR2, TGIF1, THBS1, THPO, TIMP3, TINF2, TJP2, TLR2, TLR4, TLX1, TMC6, TMC8, TMEM67, TMEM127, TMEM216, TMEM231, TNFRSF10B, TNFRSF13B, TNFRSF13C, TOP1, TP53, TP63, TREM2, TREX1, TRIM24, TRIM33, TRIM37, TRIP11, TRIP13, TRPS1, TRPV3, TRRAP, TSC1, TSC2, TSHR, TTC37, TWIST1, TXNRD2, TYR, TYROBP, UGT1A1, UROD, USB1, USP9X, VANGL1, VANGL2, VEGFC, VHL, WAS, WASHC5, WDPCP, WIPF1, WNT5A, WNT10A, WRAP53, WRN, WT1, WWOX, XIAP, XPA, XPC, XRCC2, XRCC3, XRCC4, YY1, ZAP70, ZFHX3, ZFPM2, ZIC2, HNF1B, IKBKG, KRIT1, NCOA4, RUNX1, TAF15, TERC, ICK.
